# Regulation of Hippocampal Synaptic Function by the Metabolic Hormone, Leptin: Implications for Health and Neurodegenerative Disease

**DOI:** 10.3389/fncel.2018.00340

**Published:** 2018-10-16

**Authors:** Gemma McGregor, Jenni Harvey

**Affiliations:** Systems Medicine, Ninewells Hospital and Medical School, University of Dundee, Dundee, United Kingdom

**Keywords:** leptin, hippocampus, synaptic plasticity, Alzheimer’s disease, amyloid

## Abstract

The role of the endocrine hormone leptin in controlling energy homeostasis in the hypothalamus are well documented. However the CNS targets for leptin are not restricted to the hypothalamus as a high density of leptin receptors are also expressed in several parts of the brain involved in higher cognitive functions including the hippocampus. Numerous studies have identified that in the hippocampus, leptin has cognitive enhancing actions as exogenous application of this hormone facilitates hippocampal-dependent learning and memory, whereas lack or insensitivity to leptin results in significant memory deficits. Leptin also markedly influences some of the main cellular changes that are involved in learning and memory including NMDA-receptor dependent synaptic plasticity and glutamate receptor trafficking. Like other metabolic hormones, there is a significant decline in neuronal sensitivity to leptin during the ageing process. Indeed, the capacity of leptin to modulate the functioning of hippocampal synapses is substantially reduced in aged compared to adult tissue. Clinical studies have also identified an association between circulating leptin levels and the risk of certain neurodegenerative disorders such as Alzheimer’s disease (AD). In view of this, targeting leptin and/or its receptor/signaling mechanisms may be an innovative approach for developing therapies to treat AD. In support of this, accumulating evidence indicates that leptin has cognitive enhancing and neuroprotective actions in various models of AD. Here we assess recent evidence that supports an important regulatory role for leptin at hippocampal CA1 synapses, and we discuss how age-related alterations in this hormonal system influences neurodegenerative disease.

## Leptin and Leptin Receptors

Leptin, an endocrine hormone, is the functional product of the obese (*ob*) gene. It is principally made in white adipose tissue and once secreted, the circulating levels of leptin correlate directly to body fat content (Maffei et al., 1995; Considine et al., 1996). The fasting leptin levels that circulate in the plasma generally range from 1 ng/ml to 100 ng/ml (Boden et al., 1996). Peripherally-derived leptin readily accesses the brain via a transport mechanism that is saturable and highly sensitive to triglycerides and adrenaline (Banks et al., 2004). Hypothalamic neural circuits are a key site for the central actions of leptin with the arcuate nucleus in particular being crucial for the energy regulating functions of this hormone. However, the neuronal actions of leptin extend beyond the hypothalamus, with many areas of the brain including the hippocampus and cerebral cortex expressing leptin receptors at a high density.

Leptin receptors are produced by the diabetes (*db*) gene and six isoforms of the leptin receptor, ObRa-f, exist. All the isoforms, with the exception of ObRe, are expressed at the plasma membrane, have analogous extracellular domains, but differ in the length of their intracellular domain. The intracellular domain of the short isoforms (ObRa, c, d, f) range from 30 to 40 amino acid residues in length. Contrastingly, the long isoform, ObRb incorporates an extended (302 residues) intracellular domain, which enables the full complement of leptin receptor signaling pathways to be activated by this isoform. In contrast, the short isoforms have limited capacity to signal, with only some of the Ob-Rb-driven signaling molecules being activated by the short isoforms. ObRe is a novel leptin receptor isoform as it does not have a membrane spanning region, but it readily binds leptin. Consequently, leptin binding to ObRe is thought to aid its transport in the plasma.

Leptin receptors are highly homologous to other class I cytokine receptors; a superfamily of receptors that includes interferon receptors. Leptin binding to ObR activates janus tyrosine kinase 2 (JAK2), which promotes JAK2 phosphorylation. This in turn allows particular tyrosine residues located intracellularly to be phosphorylated. This sequence of events enables a number of signaling pathways to be activated by ObRs. The principle signaling molecules that are stimulated by neuronal ObRs include the signal transduction and activator of transcription (STAT) transcription factors, phosphoinositide-3 kinase (PI 3-kinase) and mitogen-activated protein kinase (MAPK; Farooqi and O’Rahilly, 2014).

## Leptin Regulation of Hippocampal Synaptic Function

Anatomical evaluation of ObR expression in the brain has detected a high density of this receptor in different regions of the hippocampus and specifically at hippocampal synapses (Hâkansson et al., 1998; Shanley et al., 2002). In line with this expression pattern, leptin treatment potently regulates excitatory synaptic transmission evoked at hippocampal Schaffer-collateral (SC)-CA1 synapses (Shanley et al., 2001; Oomura et al., 2006; Moult et al., 2010; Moult and Harvey, 2011). Moreover, studies in obese rodents that are leptin-insensitive have also identified significant impairments in two key forms of hippocampal synaptic plasticity, namely long-term potentiation (LTP) and long-term depression (LTD). Additionally, marked deficits in behavioral assessment of hippocampal-dependent memory have also been reported in these obese rodents (Li et al., 2002; Winocur et al., 2005). Together these findings support the notion that leptin has potential cognitive enhancing actions.

**Figure 1:**
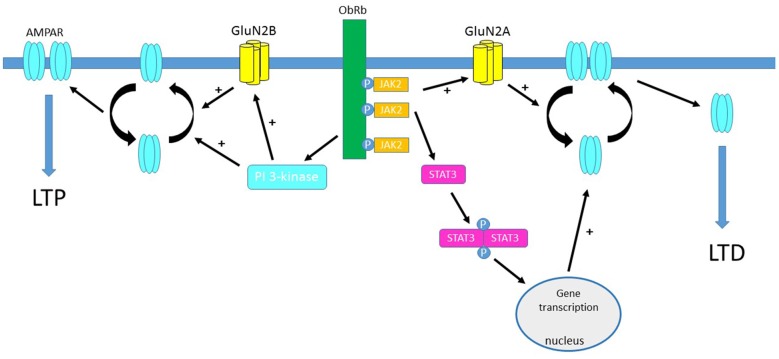
Activation of leptin receptors influences hippocampal synaptic efficacy at temporoammonic (TA)-CA1 synapses. Schematic representation of the predominant signaling pathways that are activated by leptin receptors located at hippocampal TA-CA1 synapses. Leptin binding to ObRb results in phosphorylation of janus tyrosine kinase 2 (JAK2) which in turn promotes phosphorylation and dimerization of signal transducer and activator of transcription 3 (STAT3), leading to gene transcriptional changes in the nucleus. Activation of JAK2-STAT3 signaling mediates leptin-induced AMPA receptor internalization and induction of long-term depression (LTD) at adult TA-CA1 synapses. The ability of leptin to induce LTD at adult TA-CA1 synapses also requires the activation of GluN2A-containing NMDA receptors, which also promotes the removal of AMPA receptors from hippocampal synapses. In contrast, at juvenile TA-CA1 synapses, leptin-driven stimulation of the phosphoinositide-3 kinase (PI 3-kinase) cascade, culminates in the synaptic insertion of AMPA receptors and the induction of long-term potentiation (LTP). Activation of GluN2B NMDA receptor subunits is pivotal for leptin-induced LTP and the synaptic insertion of GluA2-lacking AMPA receptors at juvenile TA-CA1 synapses.

More recent studies indicate that application of leptin also influences the magnitude of excitatory synaptic transmission at another synaptic input to CA1 neurons. This input, which arises in the entorhinal cortex (EC), forms part of the temporoammonic (TA) pathway (Luo et al., 2015; McGregor et al., 2018; Figure 1). Indeed, application of leptin to juvenile (11–18) hippocampal slices induces LTP at this synaptic connection (Luo et al., 2015), which directly contrasts with the leptin-induced depression of synaptic transmission observed at SC-CA1 synapses at this age (Moult and Harvey, 2011). Interestingly, although leptin has divergent actions at the two synaptic inputs to pyramidal neurons, activation of NMDA receptors is essential for both forms of leptin-dependent synaptic plasticity (Moult and Harvey, 2011; Luo et al., 2015). The involvement of NMDA receptors parallels the dependance on NMDA receptors for other key modulatory effects of leptin in the hippocampal formation. For instance, the ability of leptin to rapidly increase the density of hippocampal dendritic filopodia is dependent on NMDA receptor activation as the morphological changes induced by leptin are diminished in the presence of the NMDA receptor antagonist, D-AP5 (O’Malley et al., 2007). Similarly, a requirement for NMDA receptors has been demonstrated for the leptin-driven reversal of LTP (known as depotentiation) as this process is blocked following antagonism of NMDA receptors. In the thalamic-lateral amygdala pathway, NMDA receptors are also required for the depotentiation of amygdala LTP induced by leptin (Wang et al., 2015). Interestingly recent studies indicate that NMDA receptors, and specifically GluN2B-containing NMDA receptors in hypothalamic AgRP neurons, are critically involved in leptin-dependent homeostatic control of body weight (Üner et al., 2015).

## Leptin Regulation of AMPA Receptor Trafficking

Synaptic insertion and removal of AMPA receptors is known to be important for driving long term alterations in excitatory synaptic efficacy at hippocampal synapses (Collingridge et al., 2004; Herring and Nicoll, 2016). Transient changes in the molecular identity of synaptic AMPA receptors following LTP induction has been detected in some studies (Plant et al., 2006; Morita et al., 2014), but there are some exceptions (Tang et al., 1999; Clayton et al., 2002). In a similar manner, leptin promotes insertion of AMPA receptors into hippocampal synapses, and this is pivotal for leptin driven changes in synaptic efficacy (e.g., LTP) at adult hippocampal SC-CA1 synapses (Moult et al., 2010). Thus, the rectification index of synaptic AMPA receptors is enhanced by leptin, and addition of philanthotoxin, to selectively inhibit GluA2-lacking AMPA receptors, results in reversal of LTP induced by leptin. Together this suggests that incorporation of GluA2-lacking AMPA receptors into synapses is a key mechanism underlying leptin-induced LTP (Moult et al., 2010). In agreement with this, studies using a combination of immunocytochemical and biotinylation techniques have demonstrated that exposure to leptin increases the plasma membrane expression of the AMPA receptor GluA1 subunit in primary cultures of hippocampal neurons and in hippocampal slices (Moult et al., 2010).

Trafficking of AMPA receptors is also essential for the reported effects of leptin at TA-CA1 synapses (Figure 1). Indeed, application of philanthotoxin also reverses the persistent increase in synaptic transmission induced by leptin at juvenile TA-CA1 synapses, suggesting that GluA2-lacking AMPA receptor are inserted into synapses by leptin and that this process is required for leptin-induced LTP (Luo et al., 2015). Conversely, the synaptic removal of GluA2-lacking AMPA receptors is implicated in leptin-induced LTD as leptin fails to induce LTD in the presence of selective inhibitors of clathrin-mediated endocytosis (McGregor et al., 2018). In a manner similar to SC-CA1 synapses, application of leptin is not only capable of inducing LTD but this also results in a significant reduction in GluA1 surface expression in hippocampal slices (McGregor et al., 2018).

Significant evidence indicates that activity-dependent LTP and LTD is likely to be key cellular events that underlie learning and memory processes that are hippocampus-dependent (Bliss and Collingridge, 1993). Moreover, occlusion studies have demonstrated that leptin-induced changes in excitatory synaptic strength evoked at SC-CA1 and TA-CA1 synapses occlude activity-dependent synaptic plasticity, indicating similar expression mechanisms (Moult and Harvey, 2011; Luo et al., 2015; McGregor et al., 2018). Thus, as leptin induces novel forms of synaptic plasticity, and regulates AMPA receptor trafficking processes, which mirrors the cellular processes that are implicated in hippocampal-dependent learning and memory, it suggests that leptin has cognitive enhancing properties.

## Protective Actions of Leptin in the CNS

A possible protective role for leptin in the CNS was first suggested in comparative studies that identified significant brain changes in leptin deficient *ob/ob* mice compared to their wildtype littermates (Ahima et al., 1999). In these studies, the brain weight of *ob/ob* mice was markedly lower than wildtype mice suggesting that lack of leptin reduces neuronal viability (Ahima et al., 1999). In support of this, treatment of *ob/ob* mice with leptin for up to 4 weeks completely reversed the observed brain abnormalities (Ahima et al., 1999). Numerous subsequent studies support the notion that leptin has protective actions centrally. Thus studies performed in central and peripheral neurons indicate that exposure to leptin not only enhances the rate of neuronal survival, but also prevents neuronal cell death induced by a variety of apoptotic stimuli (Doherty et al., 2008; Guo et al., 2008; Davis et al., 2014).

Protective effects of leptin have been shown in several CNS-driven disease models that are associated with neuronal apoptosis. In a mouse model of cerebral ischemia, treatment with leptin decreased the extent of brain injury as the overall infarct volume was attenuated by leptin (Zhang et al., 2013). Similarly, in an oxygen/glucose deprivation model of ischemia, leptin protects against ischemic damage (Zhang and Chen, 2008). In various models of Parkinson’s disease (PD), the viability of dopaminergic neurons treated with either 6-OH dopamine or MPTP is significantly enhanced after treatment with leptin (Weng et al., 2008; Doherty et al., 2008). Moreover, prior exposure of human SH-SY5Y neuroblastoma cells with leptin also prevented neuronal cell death induced by the neurotoxin 1-methyl-4-pyridinium (MPP^+^; Lu et al., 2006).

It is interesting to note that the neuroprotective properties of metabolic hormones is not restricted to leptin. Thus, ghrelin, which is produced in the stomach and regulates body weight by promoting food intake, also markedly influences neuronal viability (de Candia and Matarese, 2018). In hypothalamic cells, treatment with ghrelin protects against oxygen-glucose deprivation and subsequent apoptosis by inhibiting mitochondrial formation of reactive oxygen species (Chung et al., 2007). The toxic apoptotic actions of Aβ are also attenuated in hypothalamic cells and hippocampal neurons following treatment with ghrelin (Moon et al., 2011; Gomes et al., 2014). Moreover, in a mouse model of traumatic brain injury, ghrelin limits the degree of neuronal degeneration by reducing apoptosis (Lopez et al., 2014). Recent evidence indicates that like leptin, age-related alterations occur in ghrelin function which are thought to lead to an increased risk of neurodegenerative disease (de Candia and Matarese, 2018). As both these metabolic hormones have comparable neuroprotective actions in the hippocampus, and given their closely related hypothalamic functions, it is feasible that there is some cross-talk between the hormones in terms of neuroprotective mechanisms. Although evidence of this is limited, leptin and ghrelin are both reported to prevent Aβ-induced cell death via inhibition of GSK-3β (Martins et al., 2013). However, further studies are required to examine potential interactions between these metabolic hormones and in turn how this impacts on CNS health.

## Leptin and Aging

It is known that as individuals get older, the functioning of metabolic hormones deteriorates and this has implications for normal CNS function as metabolic dysfunction has been linked to faster ageing and an increased likelihood of developing neurodegenerative conditions such as Alzheimer’s disease (AD; Stranahan and Mattson, 2011; Kim and Feldman, 2015). Several lines of evidence support the notion that age-related changes in the effectiveness of the leptin system occurs in the CNS. Indeed, hypothalamic neurons are less responsive to leptin with age as the satiety effects of leptin are markedly reduced in aged (30 month) compared to adult (6 month) rats (Shek and Scarpace, 2000). At the cellular level, reductions in leptin-driven activation of STAT3 as well as phosphorylated-STAT3 binding activity have been observed in aged rats (Shek and Scarpace, 2000; Scarpace et al., 2001). Attenuated uptake of leptin into the hypothalamus, as well as reductions in the capacity of ObRs to signal, due to elevations in suppressor of cytokine signaling-3 (SOCS-3), have also been reported in aged animals (Wang et al., 2001; Peralta et al., 2002).

Age-dependent changes in response to leptin have also been detected at hippocampal synapses. At juvenile (P14–21) SC-CA1 synapses, treatment of hippocampal slices with leptin markedly depresses excitatory synaptic transmission, however this effect is transient as it is readily reversed on leptin washout (Shanley et al., 2001; Xu et al., 2008; Moult and Harvey, 2011). In contrast, leptin has directly opposing actions in hippocampal slices obtained from adult (3–4 months) rats as exposure to leptin culminates in LTP induction at SC-CA1 synapses. Moreover the magnitude of leptin-induced LTP at SC-CA1 synapses is significantly altered with age, as leptin-induced LTP is attenuated by around 50% in aged (12–14 months) relative to adult tissue (Moult and Harvey, 2011). Thus, not only does leptin have bi-directional age-dependent effects on synaptic efficacy at excitatory SC-CA1 synapses, but there is also a marked reduction in the sensitivity to leptin with age.

Recent evidence indicates that the modulatory actions of leptin at excitatory TA-CA1 synapses is also highly dependent on age (McGregor et al., 2018; Figure 1; Table 1). In juvenile tissue, application of leptin leads to TA-CA1 LTP (Luo et al., 2015), whereas a persistent depression (LTD) is observed after leptin addition to adult hippocampal slices (McGregor et al., 2018). Moreover like SC-CA1 synapses, the leptin-sensitivity of TA-CA1 synapses markedly decreases in aged tissue, as application of leptin fails to induce LTD at aged TA-CA1 synapses (McGregor et al., 2018). It is unclear why leptin has no effect at aged excitatory TA-CA1 synapses. However the lack of leptin responsiveness is unlikely to be due to age-related changes in NMDA receptor expression as TA-CA1 LTP is readily induced by a high frequency stimulation protocol in slices from aged animals, and like leptin-induced LTD, the cellular mechanisms underlying TA-CA1 LTP are dependent on GluN2A-containing NMDA receptors (McGregor et al., 2018).

**Table 1:** Summary of the opposing actions of leptin at hippocampal synapses with age.

SC-CA1 synapse	TA-CA1 synapse
**Juvenile (P11–18) hippocampal slices**	
**Leptin-induced synaptic depression**	**Leptin-induced LTP**
GluN2B-dependent	GluN2B-dependent
ERK signaling	PI 3-Kinase signaling
Removel of GluA2-lacking AMPARs	Insertion of GluA2-lacking AMPARs
**Adult (3–6 months) hippocampal slices**	
**Leptin-induced LTP**	**Leptin-induced LTD**
GluN2A-dependent	GluN2A-dependent
PI 3-Kinase signaling	JAK2-STAT3 signaling
Insertion of GluA2-lacking AMPARs	Removel of GluA2-lacking AMPARs
**Aged (12–15 months) hippocampal slices**	
**Leptin-induced LTP**	**Leptin fails to induce LTD**
Magnitude of leptin-induced LTP is reduced.	LFS also fails to induce LTD

*Summary table illustrating the bi-directional effects of the hormone leptin at hippocampal Schaffer-collateral (SC)-CA1 and temporoammonic (TA)-CA1 synapses. At juvenile SC-CA1 synapses, application of leptin results in a transient depression of excitatory synaptic transmission that is not only NMDA receptor dependent but involves selective activation of GluN2B-containing NMDA receptors. In addition, activation of an ERK-dependent process and subsequent removal of GluA2-lacking AMPA receptors underlies this effect of leptin. Conversely at adult (3–6 months) SC-CA1 synapses, a novel form of long-term potentiation (LTP) is evoked by leptin. This process is GluN2A-dependent, requires activation of phosphoinositide-3 kinase (PI 3-kinase) and the synaptic insertion of GluA2-lacking AMPA receptors. The ability of leptin to regulate SC-CA1 synapses markedly declines with age as the magnitude of leptin-induced LTP at SC-CA1 synapses is significantly attenuated in aged hippocampus. At all stages of development and ageing, leptin has opposing actions on synaptic efficacy at the anatomically distinct TA input to CA1 neurons. Thus in contrast to its actions at SC-CA1 synapses, leptin induces a novel form of NMDA-dependent LTP at juvenile TA-CA1 synapses. Leptin-induced LTP involves selective activation of GluN2B subunits and PI 3-kinase-driven trafficking of GluA2-lacking AMPA receptors to synapses. In contrast, application of leptin to adult hippocampal slices leads to the induction of a novel form of NMDA receptor-dependent long-term depression (LTD) at TA-CA1 synapses. Leptin-induced LTD is GluN2A-dependent and involves activation of canonical janus tyrosine kinase 2 (JAK2)-signal transducer and activator of transcription 3 (STAT3) signaling and internalization of GluA2-lacking AMPA receptors. Like SC-CA1 synapses, there is a marked reduction in the sensitivity of TA-CA1 synapses with age, as leptin fails to induce LTD at aged TA-CA1 synapses*.

Studies examining the potential cellular mechanisms contributing to the age-dependent actions of leptin have evaluated the role of NMDA receptors, as hippocampal NMDA receptors comprising different NMDA receptor subunits are implicated in divergent types of synaptic plasticity (Liu et al., 2004; Bartlett et al., 2007), and NMDA receptor expression and molecular identity varies at different stages of development and ageing (Monyer et al., 1994). Using pharmacological tools to selectively block different GluN2 subunits, it has been shown that distinct NMDA receptor subunits contribute to the bi-directional and age-related actions of leptin at hippocampal synapses. Thus, NMDA receptors comprised of GluN2B subunits mediate the leptin-driven synaptic depression at juvenile SC-CA1 synapses. By contrast, in adult hippocampus, activation of NMDA receptors that contain GluN2A subunits is key for the induction of LTP by leptin at SC-CA1 synapses. (Moult and Harvey, 2011). At TA-CA1 synapses, NMDA receptors with distinct molecular composition are also implicated in the opposing age-dependent effects of leptin. Thus, activation of GluN2B subunits is required for leptin-induced LTP in juvenile tissue (Luo et al., 2015) whereas GluN2A subunits underlie leptin-induced LTD in adult hippocampus (McGregor et al., 2018; Table 1). The signaling pathways that link leptin receptor activation to modulation of synaptic efficacy also differ depending on age and synaptic connection. Thus, at adult SC-CA1 synapses, PI3-kinase activation is crucial for leptin-induced LTP, but stimulation of ERK-dependent signaling is essential for the synaptic depression induced by leptin at juvenile slices. Interestingly, divergent signaling pathways also mediate the age-related modulatory actions of leptin at TA-CA1 synapses as LTP induce by leptin at juvenile synapses requires GluN2B subunits and PI 3-kinase-driven signaling (Luo et al., 2015; Figure 1). However in adult, leptin-induced TA-CA1 LTD is unique as JAK2-STAT3 signaling which in turn drives gene transcriptional changes are fundamental for this form of synaptic plasticity (McGregor et al., 2018; Figure 1). Overall these data suggest that the age-dependent effects of leptin on excitatory synaptic transmission at the two distinct inputs to CA1 pyramidal neurons are dependent on the molecular composition of NMDA receptors.

## Leptin and Alzheimer’S Disease

Age significantly increases the risk of developing AD, and as a consequence the prevalence of this disease is steadily increasing as people live longer. Other prominent factors that influence AD risk, include diet and lifestyle. Indeed, mid-life obesity significantly alters the risk of AD later in life, when compared to individuals with normal body weight. Increased body fat content results in higher circulating levels of leptin and subsequent development of leptin resistance in the obese state (Friedman, 2014). Thus AD risk may also be significantly altered in individuals with resistance to leptin and/or altered responsiveness to leptin. In support of this notion, attenuated levels of leptin in the plasma are documented in AD patients (Power et al., 2001), and prospective studies have found that low leptin levels is linked to an elevated risk of AD with age (Lieb et al., 2009). The fact that very high leptin levels (and subsequent development of leptin resistance) as well as low leptin levels are both associated with an increased risk of AD suggests that divergent metabolic states can influence the risk of AD. Although these clinical findings appear to be contradictory, there are parallels to the regulatory actions of leptin in the hypothalamus. Thus, the ability of leptin to regulate food intake and body weight occurs within a tightly regulated concentration range, such that too little circulating leptin fails to influence energy homeostasis, whereas highly elevated leptin levels that occur in the obese state not only results in leptin resistance but also loss of leptin’s capacity to regulate food intake (Friedman, 2014). Overall this suggests that failure to maintain normal body weight and thus keep leptin levels within the physiological range, results in an increased risk of developing AD.

In a manner similar to AD patients, alterations in leptin function have also been observed in several rodent models of AD. Indeed transgenic models that have the same mutations that occur in familial AD, also exhibit reduced circulating leptin levels, suggesting compromised leptin function (Fewlass et al., 2004). However some clinical studies have found no apparent link between plasma leptin levels and AD. One possible reason for this discrepancy is that the plasma levels of leptin may not be a good indicator of leptin levels in the CNS. Indeed, altered blood-brain transport of leptin has been documented in AD (Dietrich et al., 2008), suggesting that the CNS levels of leptin are reduced in AD compared to normal. However, studies that have directly measured the brain levels of leptin have reported increased or unaltered leptin levels in AD patients (Bonda et al., 2014; Maioli et al., 2015). However, attenuated expression of leptin receptor mRNA and immunoreactivity is evident in post-mortem AD tissue, suggesting possible development of resistance to leptin (Bonda et al., 2014; Maioli et al., 2015). Reductions in some key signaling pathways that are activated downstream of leptin receptors have been observed in AD tissue which also supports the possibility that central resistance to leptin develops in AD (Maioli et al., 2015).

## Protective Actions of Leptin in AD Models

**Figure 2:**
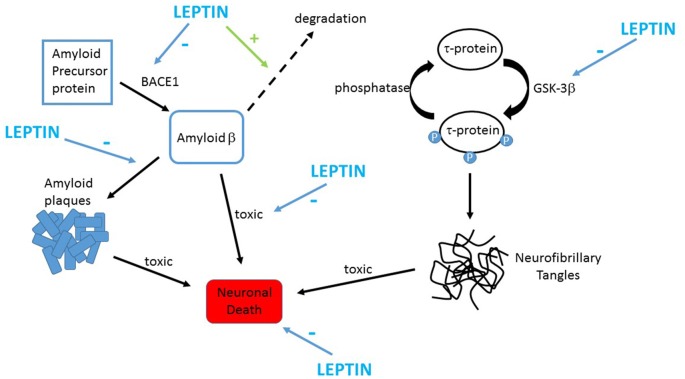
Leptin prevents the key pathological changes in Alzheimer’s disease (AD). Schematic representation of the protective actions of leptin in AD. Toxic forms of Aβ are formed by the proteolytic processing of APP by the enzyme BACE1. Leptin limits production of Aβ in neurons via inhibiting BACE1 activity. Leptin also attenuates extracellular Aβ levels by promoting the clearance and degradation of Aβ. Accumulation of Aβ promotes assembly of fibrillary forms of Aβ that leads to formation amyloid plaques. Leptin reduces plaque formation by reducing expression of GM1 gangliosides thereby limiting assembly of fibrillary Aβ. Leptin also counteracts the harmful effects of Aβ as it protects against the toxic actions of Aβ via stimulation of pro-survival signaling pathways. Hyper-phosphorylation of tau protein occurs in AD which results in formation of neurofibrillary tangles. GSK-3β drives phosphorylation of tau, and leptin limits this via inhibiting the activity of GSK-3β.

Increasing evidence indicates that leptin has protective actions in a variety of cellular systems that model the neuronal degeneration that occurs in AD. Several studies have revealed that the levels of toxic Aβ are diminished after treatment with leptin (Figure 2). Indeed, the activity of β secretase, a key enzyme involved in the production of Aβ_1–42_, is reduced by leptin resulting in decreased levels of Aβ_1–42_ (Fewlass et al., 2004). In H4 neuroglioma cells, transcription of presenilin 1 is also down-regulated by leptin which in turn leads to attenuated Aβ_1–42_ levels (Niedowicz et al., 2013). Uptake of Aβ is also modulated by leptin as LRP1-mediated uptake of Aβ is elevated after treatment of human SHSY-5Y neuroblastoma cells with leptin (Fewlass et al., 2004). Furthermore, leptin enhances the degradation of Aβ by increasing the levels of insulin degrading enzyme in organotypic brain slices (Marwarha et al., 2010).

In neurotoxicity assays, exposure of hippocampal or cortical neurons to leptin protects against the harmful actions of oligomeric Aβ on neuronal viability (Doherty et al., 2013; Martins et al., 2013; Figure 2). Leptin also prevents the increased aggregation of Aβ that occurs after treatment of cortical neurons with Cu^2+^ ions (Doherty et al., 2013). In addition, leptin is reported to have neuroprotective actions in hypothalamic cells, as the ability of oligomeric Aβ to increase superoxide production and elevate intracellular calcium levels are significantly attenuated by leptin in mHypoE-N42 cells (Gomes et al., 2014).

It is known that increased expression of the presynaptic protein, endophilin 1 occurs in those suffering from AD and in transgenic AD models (Ren et al., 2008). As endophilin 1 inhibits the synaptic release of glutamate (Weston et al., 2011), AD-linked increases in endophilin 1 are likely to have a direct impact on excitatory synaptic transmission. Recent evidence has demonstrated that exposure to leptin also alters expression of endophilin1, as leptin inhibits the increase in endophilin 1 induced by Aβ (Doherty et al., 2013). Moreover, the cortical levels of endophilin 1 are up-regulated in Zucker *fa/fa* rats which indicates that insensitivity to leptin elevates the brain levels of endophilin 1 *in vivo* (Doherty et al., 2013). Consequently it is possible, that leptin, by regulating endophilin 1 expression, indirectly influences excitatory synaptic transmission at hippocampal synapses.

In addition to preventing the chronic actions of Aβ, leptin is also capable of directly influencing the impact of acute Aβ on hippocampal synaptic function. It is well established that short-term treatment with Aβ not only results in inhibition of activity-dependent LTP, but it also enhances LTD in hippocampal slices (Shankar et al., 2008; Li et al., 2009). Moreover exposure to leptin is reported to reverse Aβ-driven inhibition of hippocampal LTP and it prevents facilitation of LTD induced by Aβ (Doherty et al., 2013; Malekizadeh et al., 2017). Direct effects of Aβ on AMPA receptor trafficking processes have also been observed, such that treatment with Aβ promotes internalization and synaptic removal of GluA1 subunits in hippocampal cultures (Li et al., 2009; Doherty et al., 2013). Prior treatment with leptin also counteracts this detrimental effect of Aβ as the ability of Aβ to internalize GluA1 subunits is blocked by leptin (Doherty et al., 2013; Malekizadeh et al., 2017). PI 3-kinase activation is required for the protective effects of leptin as pharmacological inhibition of this signaling cascade blocked the ability of leptin to prevent the acute actions of Aβ on hippocampal synapses (Doherty et al., 2013).

It has been proposed that oligomeric Aβ facilitates the induction of LTD via preventing uptake of glutamate at hippocampal synapses (Li et al., 2009). This process is likely to enhance glutamate levels within the synaptic cleft leading to activation and subsequent desensitization of synaptic NMDA receptors (Li et al., 2009). One of the proposed functions of LTD is to enhance the flexibility of networks by providing a way of re-setting potentiated synapses, thereby preventing saturation of hippocampal synapses which would limit capacity for learning. However this normal function of LTD is likely to be compromised in AD, as hippocampal synapses exposed to Aβ are unable to be potentiated/saturated due to the block of LTP induction by Aβ. Consequently in AD, hippocampal excitatory synapses are likely to remain in a depressed state; an effect that would be reinforced by sustained removal of AMPA receptors from synapses by Aβ. As treatment with leptin prevents the aberrant effects of Aβ on hippocampal LTP and LTD, as well as AMPA receptor trafficking, it is likely that restoration of normal hippocampal synaptic function would occur after treatment with leptin.

However, as leptin also has direct effects on excitatory synaptic efficacy, including its ability to induced novel forms of LTP and LTD at hippocampal synapses, do the protective actions of leptin have a bearing on its potential cognitive enhancing properties? What is clear is that the ability of leptin to prevent the acute synapto-toxic actions of Aβ occurs at low concentrations of leptin that have little or no effect of basal excitatory synaptic transmission (Doherty et al., 2013). In contrast, however, the marked effects of leptin on hippocampal synaptic plasticity that are likely to enhance cognition, occur at much higher leptin concentrations. This suggests that the protective and cognitive enhancing actions of leptin are distinct, as they have differing pharmacological profiles.

## Leptin Influences Tau-Related Pathology in AD

Neurofibrillary tangles are another major feature of AD pathology and hyper-phosphorylated tau is a key element of these tangles. Increasing evidence suggests that leptin limits accumulation of tau within the brain. Indeed, significantly lower phosphorylated tau (p-tau) levels are detected in TgCRND8 mice, that overexpress mutant human APP, after treatment with leptin (Greco et al., 2010). Tau phosphorylation status is also directly reduced by leptin as the activity of GSK3β, a key enzyme that drives tau phosphorylation, is inhibited by this hormone (Greco et al., 2009, 2010; Figure 2). Cortical neurons that are chronically treated with Aβ also exhibit elevated levels of p-tau and this process is markedly reduced by leptin (Doherty et al., 2013). In addition, impaired leptin function is associated with alterations in cortical p-tau levels *in vivo* as markedly elevated levels of p-tau have been detected in leptin-insensitive Zucker *fa/fa* rats (Doherty et al., 2013). Moreover, development of leptin resistance has recently been associated with enhanced tau pathology in mouse models of AD (Platt et al., 2016). Thus it is clear that leptin not only limits phosphorylation of tau, but also that lack of leptin receptor-driven signaling increases p-tau levels which lends support to the hypothesis that AD risk is influenced by impairments in the leptin system (Power et al., 2001; Lieb et al., 2009).

## Leptin Improves Memory in AD Models

Significant evidence indicates that activity-dependent LTP and LTD are likely to be key cellular events that underlie learning and memory processes that are hippocampus-dependent (Bliss and Collingridge, 1993). Moreover, occlusion studies have demonstrated that leptin-driven changes in excitatory synaptic strength evoked at SC-CA1 and TA-CA1 synapses occlude activity-dependent synaptic plasticity, indicating similar expression mechanisms (Moult and Harvey, 2011; Luo et al., 2015; McGregor et al., 2018). Thus, as leptin induces novel forms of synaptic plasticity, and it potently regulates AMPA receptor trafficking processes, both of which mirror the cellular processes that are implicated in hippocampal-dependent learning and memory, it suggests that leptin has cognitive enhancing properties. Indeed, this is backed up in numerous behavioral studies which demonstrate that leptin has cognitive enhancing effects in rodents. Indeed intravenous administration of leptin facilitates performance in hippocampal spatial memory tests assessed using the Morris water-maze (Oomura et al., 2006). Conversely, deficits in hippocampal-dependent spatial memory are observed in leptin-insensitive rodents, suggesting that insensitivity to leptin markedly influences hippocampal-dependent memory (Li et al., 2002; Winocur et al., 2005). Diet-induced leptin resistance also interferes with memory consolidation in rats, such that marked deficits are observed in object recognition tests in diet-induced obese rats compared to wild type mice with normal body weight (Zanini et al., 2017).

Application of leptin also enhances performance in many hippocampal-dependent memory tasks in rodent models of AD. Thus, in SAMP8 mice which have elevated levels of Aβ and hippocampal memory deficits, administration of leptin improves performance in a T-maze task (Farr et al., 2006). In TgCRND8 mice, a transgenic line derived from an APP Swedish mutation that develop amyloid plaques in the hippocampus and cortex from 9 weeks of age, treatment with leptin enhances the ability to perform behavioral tests of novel object recognition and fear conditioning (Greco et al., 2010). Hippocampal memory deficits in APP/PS1 mice have also been reversed by intracerebroventricular (ICV) administration of a lentiviral vector expressing leptin (Pérez-González et al., 2014). Recent *in vivo* studies also support the notion that leptin counteracts the harmful actions of Aβ on hippocampal-dependent memory, as the spatial memory deficits induced by ICV application of Aβ(1–42) are alleviated in rats exposed to leptin (Tong et al., 2015). Thus, there is clear evidence that treatment with leptin enhances cognition in both healthy animals and in models of AD.

## The Therapeutic Potential of the Leptin System in AD

Although leptin has cognitive enhancing and neuroprotective actions in rodents, it is key for future development of leptin as an AD therapy, that its central actions in humans are well defined. Several studies have observed cognitive enhancing actions of leptin in human clinical studies. Thus, significant increases in gray matter volume have been detected in adults with congenital leptin deficiencies following treatment with physiological levels of leptin (Matochik et al., 2005). Leptin replacement therapy has also led to a significant improvement in cognitive function in a 5 year old with leptin deficiency due to a rare *ob* gene mutation (Paz-Filho et al., 2008). Although chronic leptin replacement therapy is safe and well tolerated in humans (Paz-Filho et al., 2015), there have been no clinical trials carried out to examine whether leptin is beneficial in AD patients.

However, several key factors need to be taken into account when using leptin in future clinical studies. Thus identifying which individuals are most likely to respond to leptin, and when in the disease process a leptin-based therapy will be beneficial is key. For instance, identifying individuals who have developed resistance to leptin, as a consequence of midlife obesity, may be pertinent as these individuals may be unresponsive to leptin-based therapies. In contrast, leptin treatment may be highly beneficial in AD patients with low baseline leptin levels. As leptin receptors are expressed throughout the brain, it is also vital that steps are taken to limit potential CNS side effects associated with a leptin-based therapy. In this respect, modification of the leptin molecule may enable specific targeting of the key brain regions affected in AD and thus enhance therapeutic efficacy. In support of this approach, a recombinant methionyl human form of leptin (metreleptin) has recently been developed, and gained FDA approval for use in treating lipodystrophy (Paz-Filho et al., 2015). Several studies have found that the whole leptin molecule is not required as leptin fragments have reported biological activity and can replicate the hypothalamic actions of leptin (Grasso et al., 1997; Rozhavskaya-Arena et al., 2000). Consequently, another approach may be to develop small leptin-like molecules. It would have to be established that such leptin mimetics replicate the full spectrum of cognitive enhancing and protective actions of leptin. Moreover, retention of key properties of the whole leptin molecule such as brain penetrability will be fundamental for the future success of a leptin-based mimetic. However recent studies in rodents have produced extremely promising results as one leptin fragment, namely leptin_116–130_ was found to replicate leptin action as it displayed powerful protective effects on hippocampal synaptic function (Malekizadeh et al., 2017). Indeed, leptin_116–130_ prevented the ability of Aβ to interfere with hippocampal synaptic plasticity and promote neuronal toxicity. Moreover, peripheral administration of leptin_116–130_ replicated leptin’s cognitive enhancing properties as it improved the ability of mice to performance specific episodic-like memory behavioral tasks (Malekizadeh et al., 2017). This indicates not only that the leptin_116–130_ fragment readily enters the brain but that it also reaches the hippocampus where it markedly influences synaptic physiology and function.

## A Role for Leptin in Other Neurodegenerative Disorders?

In a manner similar to AD, increasing evidence indicates that metabolic dysfunction is a prominent feature in several other neurodegenerative diseases. Indeed, a link has been identified between mid-life obesity and the risk and progression of PD (Abbott et al., 2002; Procaccini et al., 2016). Alterations in metabolic function related to mid-life obesity are also associated with an increased likelihood of developing vascular dementia (Kivipelto et al., 2005) and Huntington’s disease (HD; Gaba et al., 2005; Procaccini et al., 2016). Clinical studies have revealed almost a two fold increase in the incidence of multiple sclerosis in obese (BMI >30) children compared to those of normal body weight (Munger et al., 2009). Several studies have observed alterations in the circulating levels of leptin in both patients and in models of these CNS disorders. Indeed, α-synuclein A53T transgenic mice, that model familial PD, exhibit metabolic abnormalities and hypoleptinemia (Rothman et al., 2014). In addition attenuated plasma levels of leptin have been detected in HD (Pratley et al., 2000; Popovic et al., 2004), and PD patients (Evidente et al., 2001).

Several studies have found that an obese phenotype exacerbates neurodegeneration in various disease models. For instance, leptin deficient *ob/ob* mice treated with either meth-amphetamine or kainic acid display significantly greater neurotoxic damage and mortality rates than lean control mice (Sriram et al., 2002). Moreover in a PD model, exposure to the neurotoxin MPTP triggers far greater degeneration of dopaminergic neurons in overfed obese mice than lean littermates fed a normal diet (Choi et al., 2005). Degeneration of dopamine neurons induced by central infusion of 6-OHDA is also significantly elevated in diet-induced obese rodents relative to lean littermates (Morris et al., 2010). Together this suggests not only that leptin dysfunction contributes to disease pathology, but also that the leptin system may be a novel target for treatment of other neurodegenerative diseases (Evidente et al., 2001; Aziz et al., 2007; Procaccini et al., 2016). However further work is required to evaluate fully the potential use of the leptin system in treating these CNS diseases.

## Conclusion

It is well documented that the hippocampus is a key CNS target for the hormone leptin and that the potential cognitive enhancing effects of leptin are due to its actions at hippocampal synapses. An established role for leptin is in its ability to regulate synaptic efficacy at SC-CA1 synapses. However, recent evidence indicates that the TA input to hippocampal CA1 neurons is also an important functional target for this hormone. The regulatory actions of leptin at excitatory TA-CA1 synapses is important as the TA pathway is an early site for degeneration in AD, and clinical studies demonstrate a link between leptin and an increased risk of AD. In addition, treatment with leptin and specific fragments of the whole leptin peptide have advantageous effects in a range of systems that model AD, suggesting that the leptin system is novel therapeutic target in AD. Although there may be limitations to the therapeutic use of leptin, due to development of leptin resistance in some individuals, it is clear that clinical studies are needed to enable thorough assessment of the therapeutic potential of the leptin system in AD patients. As the risk of developing several other neurodegenerative disorders is also linked to altered leptin function, it is feasible that targeting the leptin system may also offer possible therapeutic benefit in these CNS diseases.

## Author Contributions

JH and GM both contributed to writing the review.

## Conflict of Interest Statement

The authors declare that the research was conducted in the absence of any commercial or financial relationships that could be construed as a potential conflict of interest.
